# pH Modification of High-Concentrated Collagen Bioinks as a Factor Affecting Cell Viability, Mechanical Properties, and Printability

**DOI:** 10.3390/gels7040252

**Published:** 2021-12-07

**Authors:** Jana Stepanovska, Martin Otahal, Karel Hanzalek, Monika Supova, Roman Matejka

**Affiliations:** 1 Department of Biomedical Technology, Faculty of Biomedical Engineering, Czech Technical University in Prague, Sitna 3105, 272 01 Kladno, Czech Republic; jana.stepanovska@fbmi.cvut.cz (J.S.); karel.hanzalek@fbmi.cvut.cz (K.H.); 2 Department of Natural Sciences, Faculty of Biomedical Engineering, Czech Technical University in Prague, Sitna 3105, 272 01 Kladno, Czech Republic; martin.otahal@fbmi.cvut.cz; 3 Department of Composites and Carbon Materials, Institute of Rock Structure and Mechanics, Czech Academy of Sciences, 182 09 Prague, Czech Republic; supova@irsm.cas.cz

**Keywords:** bioprinting, bioink, collagen hydrogels, biofabrication, stromal cells, compressive elastic modulus

## Abstract

The 3D bioprinting of cell-incorporated gels is a promising direction in tissue engineering applications. Collagen-based hydrogels, due to their similarity to extracellular matrix tissue, can be a good candidate for bioink and 3D bioprinting applications. However, low hydrogel concentrations of hydrogel (<10 mg/mL) provide insufficient structural support and, in highly concentrated gels, cell proliferation is reduced. In this study, we showed that it is possible to print highly concentrated collagen hydrogels with incorporated cells, where the viability of the cells in the gel remains very good. This can be achieved simply by optimizing the properties of the bioink, particularly the gel composition and pH modification, as well as by optimizing the printing parameters. The bioink composed of porcine collagen hydrogel with a collagen concentration of 20 mg/mL was tested, while the final bioink collagen concentration was 10 mg/mL. This bioink was modified with 0, 5, 9, 13, 17 and 20 μL/mL of 1M NaOH solution, which affected the resulting pH and gelling time. Cylindrical samples based on the given bioink, with the incorporation of porcine adipose-derived stromal cells, were printed with a custom 3D bioprinter. These constructs were cultivated in static conditions for 6 h, and 3 and 5 days. Cell viability and morphology were evaluated. Mechanical properties were evaluated by means of a compression test. Our results showed that optimal composition and the addition of 13 μL NaOH per mL of bioink adjusted the pH of the bioink enough to allow cells to grow and divide. This modification also contributed to a higher elastic modulus, making it possible to print structures up to several millimeters with sufficient mechanical resistance. We optimized the bioprinter parameters for printing low-viscosity bioinks. With this experiment, we showed that a high concentration of collagen gels may not be a limiting factor for cell proliferation.

## 1. Introduction

Bioprinting of cell-incorporated gels is a promising direction for producing “customized” tissues and organs for patients. Polymers used for bioprinting vary in their properties and, therefore, suitability for this application [1]. The ideal polymer should achieve high mechanical integrity and stability, insolubility in culture medium and ideal biodegradability, while being non-toxic to cells, non-immunogenic and promoting cell adhesion and proliferation [2]. In addition, ease of workability, suitability for bioprinting, affordability and commercial availability are essential for selecting a suitable gel [2]. Synthetic gels are characterized by excellent mechanical resistance, whereas natural gels, on the other hand, achieve exceptionally high biocompatibility [3]. Therefore, it is optimal to use natural gels with a high concentration of polymers, which improves their mechanical properties [4]. Natural hydrogels such as collagen (COL), gelatine (GEL), fibrin, chitosan and alginate [5], belong to the most common type of bioink. Collagen is one of the naturally occurring polymers that are most often obtained from porcine, murine, bovine or marine connective tissue, as it is the main component of the extracellular matrix [6].

Hydrogel properties such as formation, viscosity and texture are determined mainly by the structure, molecular size, temperature and pH of the system [7]. During COL gelation, there is a lag phase where the primary aggregates of COL molecules are nucleated. Then, microfibrillar aggregation starts with the lateral aggregation of subunits, induced by changes in ionic strength and pH, and the raising of the temperature up to 37 °C, until the equilibrium is reached. In contrast, the basic mechanism of GEL is related to the reverse coil-to-helix transition triggered by cooling solutions below 30 °C, during which the helices that are created are similar to the COL triple-helix, but do not reach equilibrium. The gelation processes for both COL and GEL are thermo-reversible, but in opposite directions: COL gels melt by lowering the temperature, while GEL gels melt by raising the temperature [8]. Tuned design and fabrication of COL hydrogels can be used to reliably and reproducibly mimic a variety of soft tissues. Fabrication parameters were varied within biologically relevant ranges and included COL concentration, polymerization pH and polymerization temperature [9].

Although hydrogels have excellent biocompatibility and biodegradability properties, they are not mechanically stable. The stiffness of the hydrogels can be modified by their physical or chemical crosslinking [10,11].

Physical crosslinking methods include heating, drying, and irradiation. High temperature crosslinking of COL (>90 °C) may cause denaturation of the structure [12]. The UV-induced crosslinking reaction requires the addition of photoactivatable reagents. Chemical crosslinking also requires a chemical agent that can interact with collagen functional groups and leads to the formation of crosslinks between individual collagen molecules. The most widely used crosslinking agents are aldehydes and carbodiimides. Their use demands the proper elimination of unreacted crosslinkers and side by-products, otherwise such crosslinked COL can be connected with worse biocompatibility. The strength of natural hydrogels can be further increased by adding natural substances such as genipin or enzymes (e.g., transglutaminase TG2) that cause gel crosslinking, since hydrogel polymerization by changes in pH and temperature is not sufficient [13]. In addition, the use of genipin and enzymes is expensive.

The physical properties of the 3D cell microenvironment, such as stiffness, can influence cell growth and commitment [14]. Their structure tends to be contracted under the traction forces of the embedded cells [15]. The results of Lotz et al. [16] indicated that the crosslinking of the COL hydrogel had a reduced contraction. However, the final stiffness can be influenced by the cells encapsulated in the gels and by the new extracellular matrix produced by these cells under dynamic conditions [17]. Several studies [14,18,19] found that undifferentiated and differentiated cells responded differently to the stiffness of their environment and to collagen concentration. Cell seeding density was shown to be critical in the survival and proliferation of encapsulated cells. Generally, it can be stated that modification of the hydrogels by other components can affect the biocompatibility of the gel and cell toxicity, thereby negatively affecting the resulting cell survival percentage [20].

Collagen-based hydrogels are often used because of their similarity to extracellular matrix tissue, particularly in terms of elastic properties and composition. Thus, they are suitable for use as a three-dimensional scaffold for cells in various fields of tissue engineering, including bioprinting [21]. Collagen hydrogels at low concentrations (<10 mg/mL), which are commonly used for culture preparation, provide insufficient structural support [22]. However, in the case of gel crosslinking under changes in temperature and pH, the use of high concentrations of collagen (at least 5 mg/mL) is rare. According to a systematic review published earlier in our article [23], only the study of Ren [24] (10 mg/mL) used concentrations higher than 5 mg/mL. Other studies used the addition of additives for collagen crosslinking, e.g., Diamantidese’s study [25] used concentrations of 4, 8 and 12 mg/mL with riboflavin as the crosslinking agent, Moncal [26] used 3 and 6 mg/mL, respectively, of Pluronic F127, and Rhee el al. [27] used concentrations of 12, 15 and 17.5, but did not specify the crosslinking method. The articles often specify the concentration of collagen in the hydrogel before mixing the bioink itself, so the real concentration is lower. Low-concentration gels with high cell densities are susceptible to shrinkage, that is, uncontrollable contraction of the gel with cells [28]. The use of higher concentrations of collagen in the hydrogel is sporadic, mainly due to the limitation of cell proliferation [3,29], as well as the limitation of the ability to differentiate and diffuse waste products [30]. Thus, there are very few studies that address cell growth and differentiation in highly concentrated hydrogels. However, cell viability is influenced by more factors than just the concentration of the polymer in the hydrogel. Both the properties of the bioink itself (composition, pH, temperature, viscosity, cell culture concentration and crosslinking method) and the parameters of the bioink itself (printer system parts temperature, pressure and speed) are essential because they have a significant influence on the resulting structural strength and viability of the incorporated cells.

It is important to tailor the printing protocol both for the type of bioink, especially with regard to its properties, and for the bioprinting technology used to create the structures. Three types of bioprinting are distinguished in terms of their bioprinting method: microextrusion printing, inkjet and laser-assisted bioprinting (LaBP) [31]. A detailed description of the methods, including the effect of the parameters on printing, is given in our recent systematic review [23]. Due to the relative ease of implementation, collagen nozzle printing is most commonly used and is present in both the extrusion and inkjet methods [23]. These methods are also characterized as relatively suitable for printing hydrogels with incorporated cells, as the cells do not come into contact with cytotoxic materials [32]. On the other hand, high collagen concentration or high cell density can cause nozzle clogging [33]. This is eliminated with LaBP methods, which also achieve much better resolution [34]. Thus, due to the different needs of all technologies, the printing and bioink parameters must be optimized for a satisfactory result. In our work, we want to show that by simply modifying the pH of the bioink and adjusting the printing parameters, it is possible to create a 3D construct with incorporated cells that achieves good proliferation and high survival rates with simple physical crosslinking achieved via changes in pH and temperature, where the resulting structure is sufficiently solid and suitable for use in bioprinting.

## 2. Results and Discussion

### 2.1. Porcine Collagen Properties

The composition of isolated COL is introduced in Table 1. COL lyophilizate contains protein, water and impurities such as lipids and glycosaminoglycans. The total protein content, represented by AAs, was 59.20 by weight. The AAs distribution was defined as the number of specific AAs residues in 1000 AA units. The hydroxyproline (Hyp) content was determined as 5.63 wt.%; in addition, Proline (Pro) and Hyp belonged to the most significant AAs because their residues helped to stabilize the triple-helix of COL. The Hyp content was reflected in the degree of hydroxylation (40%), which is defined as the ratio of Hyp/(Hyp + Pro). In addition, the Pro and Hyp residues presented sites for the carbohydrate attachments, including GAGs and lipids, determined to be 4.24 and close to 24 wt.%, respectively. The high scatter of data determined in the lipid content could be connected with local inhomogeneity. The amount of interstitial water slightly exceeded 7 wt.%.

**Figure 1:**
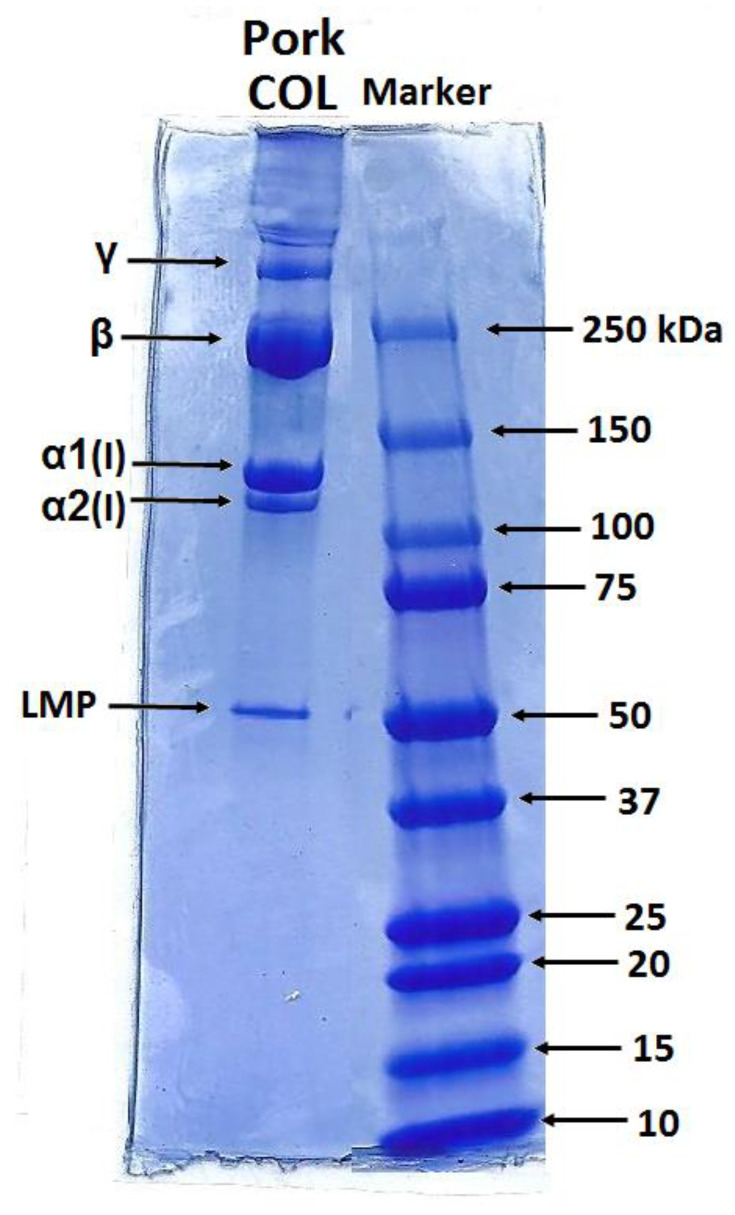
Polypeptide pattern of type COL I isolated from porcine skin.

Figure 1 shows the electrophoretic profiles of the porcine collagen. Protein patterns, comprising α1 and α2 chains with an average molecular weight of 130–110 kDa, and their dimer (β chain) and trimer (γ chain) forms, are shown. The band intensity ratio of the α1 and α2 chains was approximately 2:1, suggesting that isolated COL comprised type I, which existed in two configurations: α1(I)α2(I)α3(I) and [α1(I)]2α2(I) [35]. The electrophoretic mobility of the α1chain and α3chain was similar and, therefore, they were unable to isolate under electrophoretic conditions [36]. The band with higher molecular weight ~250 kDa belonged to β-component and was revealed to be the main component of the SDS-PAGE profile. The visible band over 250 kDa could be attributed to γ chain. On the other side, the profile also comprised lower molecular peptide (LMP) components visible at 50 kDa.

**Figure 2:**
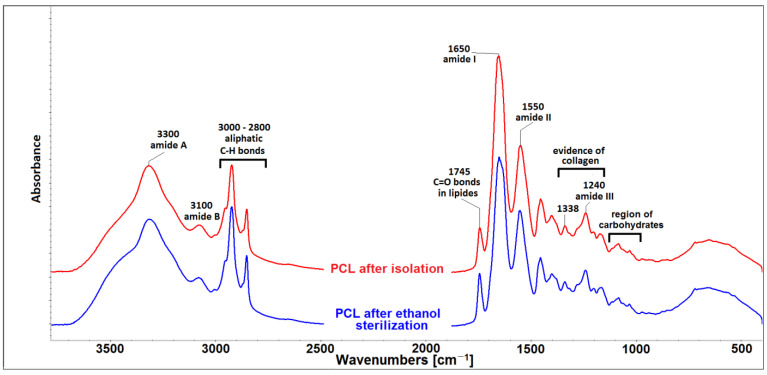
FTIR spectrum of porcine collagen lyophilisate (PCL) after isolation and after ethanol sterilization.

The FTIR spectrum of isolated COL and COL submitted to ethanol sterilization can be seen in Figure 2. The spectrum contained five amidic bands. The peak, centered at 3330 cm^−1^, comprised a mutual band of hydrogen bonds from the intermolecular water and amide A of collagen, which was associated with N-H stretching. The band at ∼3100 cm^−1^, quoted as amide B, is a common band for C–H stretching in the sp2 hybridization and stretching vibration of the N-H bonds in secondary amides. Generally, amide I bands (∼1650 cm^−1^) originate from C=O stretching vibrations coupled with N–H bending vibration. Amide II bands (∼1550 cm^−1^) arise from N–H bending vibrations coupled with C–N stretching vibrations [37]. Another source of evidence of the existence of a triple helical structure is the presence of a quartet of bands at ∼1205, 1240, 1280 (amide III) and 1338 cm^−1^ [38]. Bands in spectral region 2800–2900 cm^−1^ belonged to C-H aliphatic bonds and the bands at 1745 cm^−1^, ascribed to the C=O bonds in esters, were related to the presence of lipids. The different intensities of the lipid bands in both materials were caused by different lipid concentrations in both materials. Low intensity bands in spectral region of 990–1100 cm^−1^ belonged to carbohydrates, which were represented by GAGs. Both lipids and GAGs are residual impurities remaining in collagen after the isolation procedure [39]. Changes in the positions and intensities of individual amide bands could be associated with changes in the secondary structure of collagen. As shown in Figure 2, the use of ethanol as a sterilization agent did not change the secondary structure of COL.

Analytical methods proved that the isolated materials were based on collagen with the presence of lipids, GAGs and LMP. These peptides were found to have a lower molecular weight and shorter chain lengths, and were much more hydrophilic than the charged amide and carboxyl groups. As proven by Abdollahi et al. [40], high amounts of interactions between these peptides with water can stabilize water in a protein gel matrix and improve the water holding capacity.

### 2.2. Bioink Composition and pH Balance

**Figure 3:**
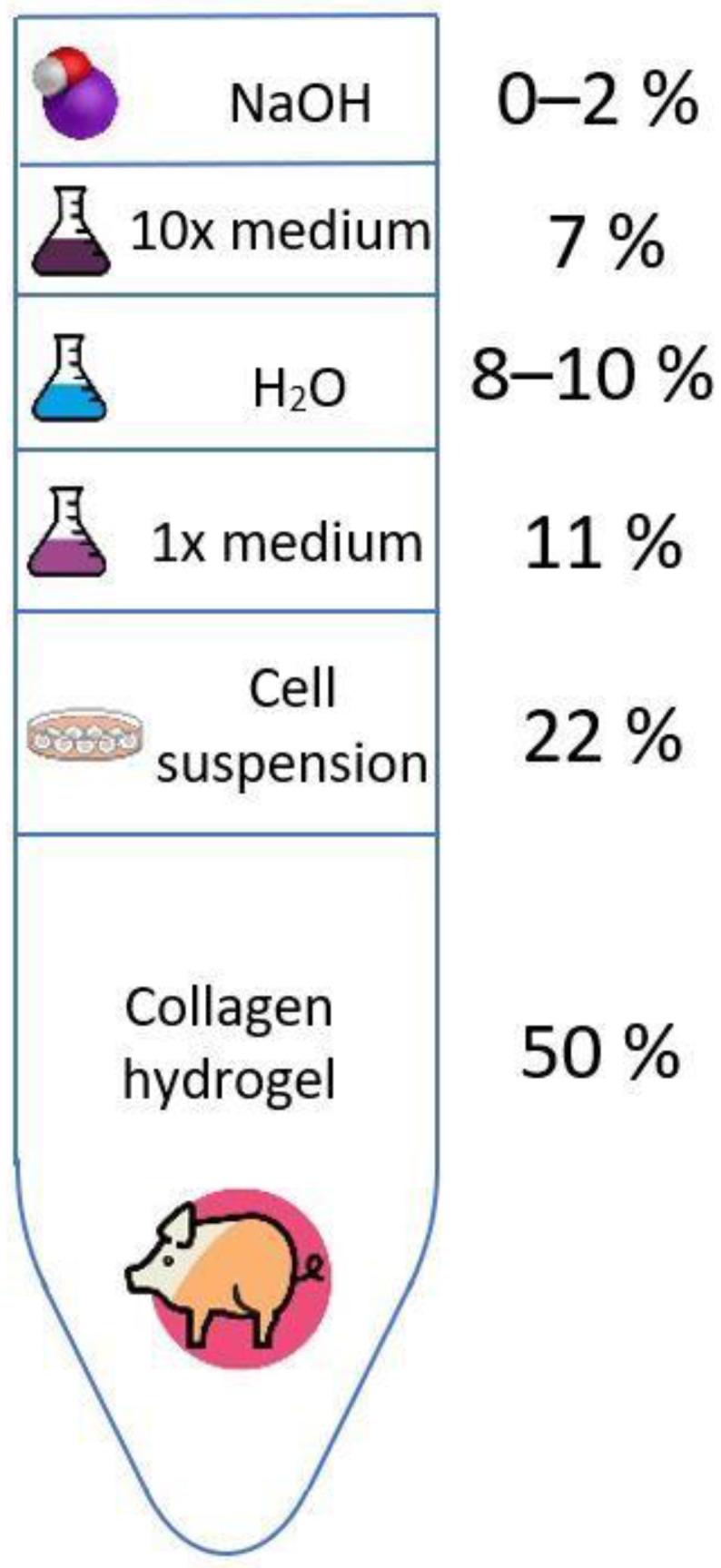
Composition of bioinks for bioprinting. The volume concentration of the 1 molar NaOH solution was varied to achieve different pH values. The water volume was then chosen so that the resulting bioink volume was always identical. More details are described in the text.

An optimal composition of collagen bioink was found, which had a balanced content of substances for cell growth and differentiation even with a high proportion of polymer in the gel. The base gel consisted of 10× concentrated medium, ultrapure water, 1× concentrated medium and collagen hydrogel. We used a collagen concentration of 20 mg/mL in the hydrogel, and the proportion of collagen in the gel was 50%, so the final concentration was then 10 mg/mL. However, this protocol is universal for use with any other initial concentration, which is reduced by 50% by mixing. Next, the pH of the gel was adjusted by adding 1M NaOH. A NaOH concentration range of 0, 5, 9, 13, 17 and 20 μL/mL was used to find the optimal pH of the hydrogel. As the following analyses show, a NaOH concentration of 13 μL/mL was found to be suitable in terms of printing and cell proliferation. After mixing all these components, an abundant gel was formed, to which—only after pH equilibration—a suspension of cells mixed in 1x concentrated medium was added. For the cells to be viable and the gel o be printable, it was necessary to maintain the proportion of components, as shown in Figure 3. The detailed composition of the gel is described in the Materials and Methods section.

**Figure 4:**
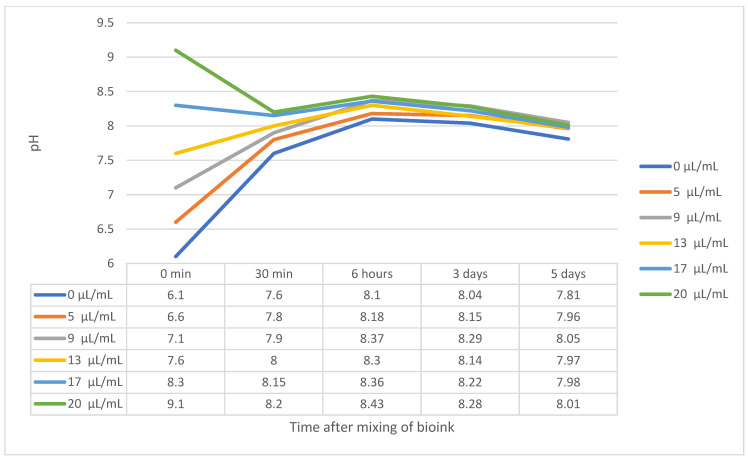
Sample pH drift. Immediately after mixing the bioink, the pH ranged from 6.1 (sample 0) to 9.1 (sample 20). However, after crosslinking the bioinks, the values became more and more alkaline, and the difference between the extremes was not so high anymore. After 3 days of cultivation, the gels became more acidic due to cell metabolism. The pH differences between the samples, especially after 5 days of cultivation, were very small and statistically insignificant. This phenomenon was caused by buffers (NaHCO_3_) in the culture medium, which was a component of the bioinks.

Due to the phenol red in the culture media, the colour of the produced bioinks greatly varied, which was particularly evident in the 0 μL/mL NaOH sample, where both the bioink and the resulting disc, before crosslinking, were coloured yellow. As the NaOH concentration increased, the samples turned from pink to purple. This colouration was indicative of the increasing pH of the samples, which was confirmed by subsequent measurement with a pH touch electrode. Immediately after bioink mixing, the pH value ranged from 6.1 for sample 0 to 9.1 for sample 20. However, due to the buffers present in the culture medium in the bioink (NaHCO_3_), the pH value approached slightly basic values after 30 min. These values were not so different; the pH of sample 0 was 7.6, and that of sample 20 was 8.2. During the following days, the gels became slightly more acidic due to cell metabolism. However, the differences between the gels were not so significant. The pH drift of all samples is shown in Figure 4.

### 2.3. The Bioprinting Procedure and Sample Form

**Figure 5:**
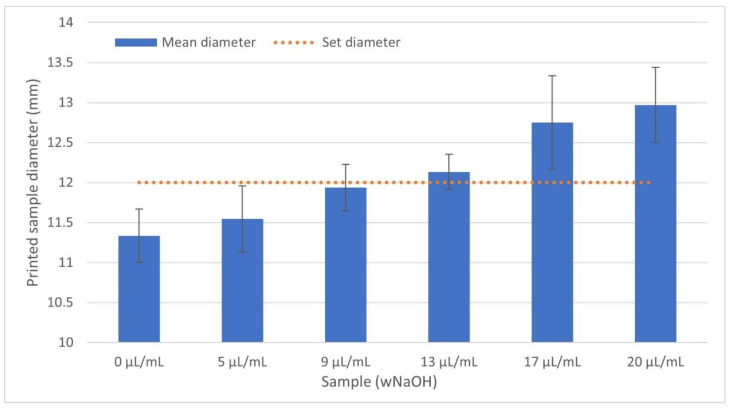
There was a difference in the final diameter of the printed cylinders depending on the concentrations of NaOH. The bars represent the mean value with SD; N = 4.

**Figure 6:**
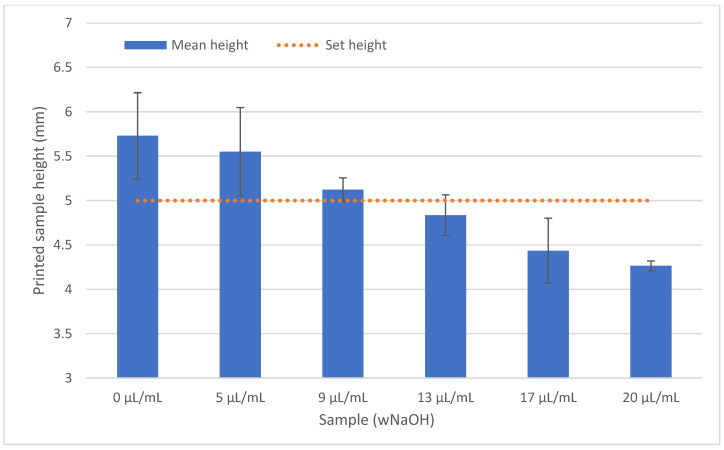
The height of the samples, depending on NaOH concentration, was negatively correlated to their diameter. The bars represent the mean value with SD; N = 4.

All produced bioink samples were homogeneous when printed, forming a regular line of circular diameter from which the resulting discs were formed. However, because of the different behaviour of the samples during printing, it was necessary to adjust the printing parameters to ensure that the line did not melt and even that the printed disc did not collapse and spill into the surroundings. As the pH of the gel increased, it was necessary to reduce the printing speed to allow the individual layers to solidify on the heated platform. Of course, this also increased the printing time. After completion, the samples were placed in an incubator for 30 min for complete crosslinking. By adjusting the printing parameters, we obtained rigid discs with dimensions close to diameter of 12 mm with height of 5 mm (smaller discs diameter of 8 mm with height of 2 mm in each case), although the viscosity of the mixed bioink varied significantly. For comparison of all samples, the same volume of bioink was used for printing. However, some dimensional changes were observable. A lower concentration of NaOH tended to cause faster gelling of the prepared samples. With the increasing of the concentration of NaOH, the printing speed was lowered; however, the samples had a diameter larger (and lower height) than that set in the model because of spilling before becoming solid. These variations are illustrated in Figure 5 and Figure 6.

The printability of a hydrogel depends on several factors related to both the printing parameters and the properties of the bioink itself. Bioink viscosity is a crucial parameter for bioprinting, but is influenced by other factors, in particular collagen concentration, cell concentration, temperature and acidity [23]. However, it is not possible to determine the appropriate viscosity for optimal printing; instead, the printing parameters must be adjusted to the viscosity of the hydrogel.

**Figure 7:**
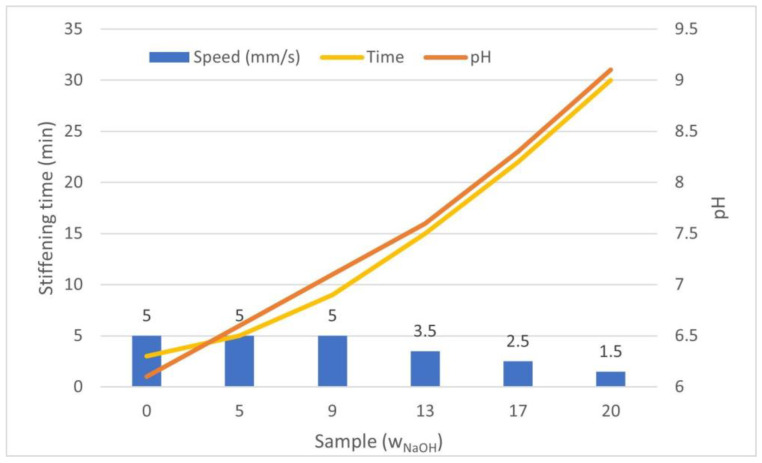
Increasing the pH of the samples caused a lower viscosity of the bioink, which then took longer to solidify. For this reason, the printing speed needed to be regulated as shown.

Parameters such as nozzle temperature, platform temperature, print speed and nozzle size mainly affect the printing result. These also contribute to the accuracy of the resulting model [41]. However, due to the relatively high density and size of molecules in concentrated collagen gels (>10 mg/mL), and the high density of cells, precision control is very limited. Finer nozzles lead to more accurate prints. We found that the use of nozzles with a diameter of less than 1 mm led to nozzle clogging, and if this led to extrusion, the printed filament would be discontinuous. With a larger nozzle diameter, more material was extruded, which may not be able to be solidified; in these cases, the speed needed to be optimized. Similarly, the pH of the bioink needed to be changed. Higher pH of collagen gels led to lower viscosity, resulting in less stability of the printed structure. If the speed was too high, the layers did not solidify enough and the structure collapsed when printing multiple layers. Therefore, the settings of our bioprinter had to be changed for individual prints. Figure 7 shows the dependence of pH on the printing speed and the approximate stiffening time of the individual samples. Although the printing speed was reduced with the increasing of the NaOH concentration, there was still an error between the set dimensions and the final dimensions of the model, as illustrated in Figure 5 and Figure 6.

The effect of heat transfer on the print was crucial, and in general, the setup where the syringe and nozzle were cooled with water at about 4 °C resulted in a 10 °C temperature of the extruder head while the platform was circulated at about 40 °C; therefore, 37 °C proved to be the best temperature for the samples. This is supported by the fact that gel crosslinking occurs as the temperature increases and the pH changes (increases to alkaline values) and is fastest around 37 °C [23]. On the contrary, not maintaining a low gel temperature in the syringe leads to solidification before printing. Under optimal conditions, especially heating the resulting structure to 37 °C, it is theoretically possible to create a structure up to several tens of millimetres high. However, as discussed below, without nutrient supply and waste removal, cell viability in structures larger than 2 mm is almost zero due to the slow diffusion of substances through the material.

### 2.4. Cell Morphology and Viability

**Figure 8:**
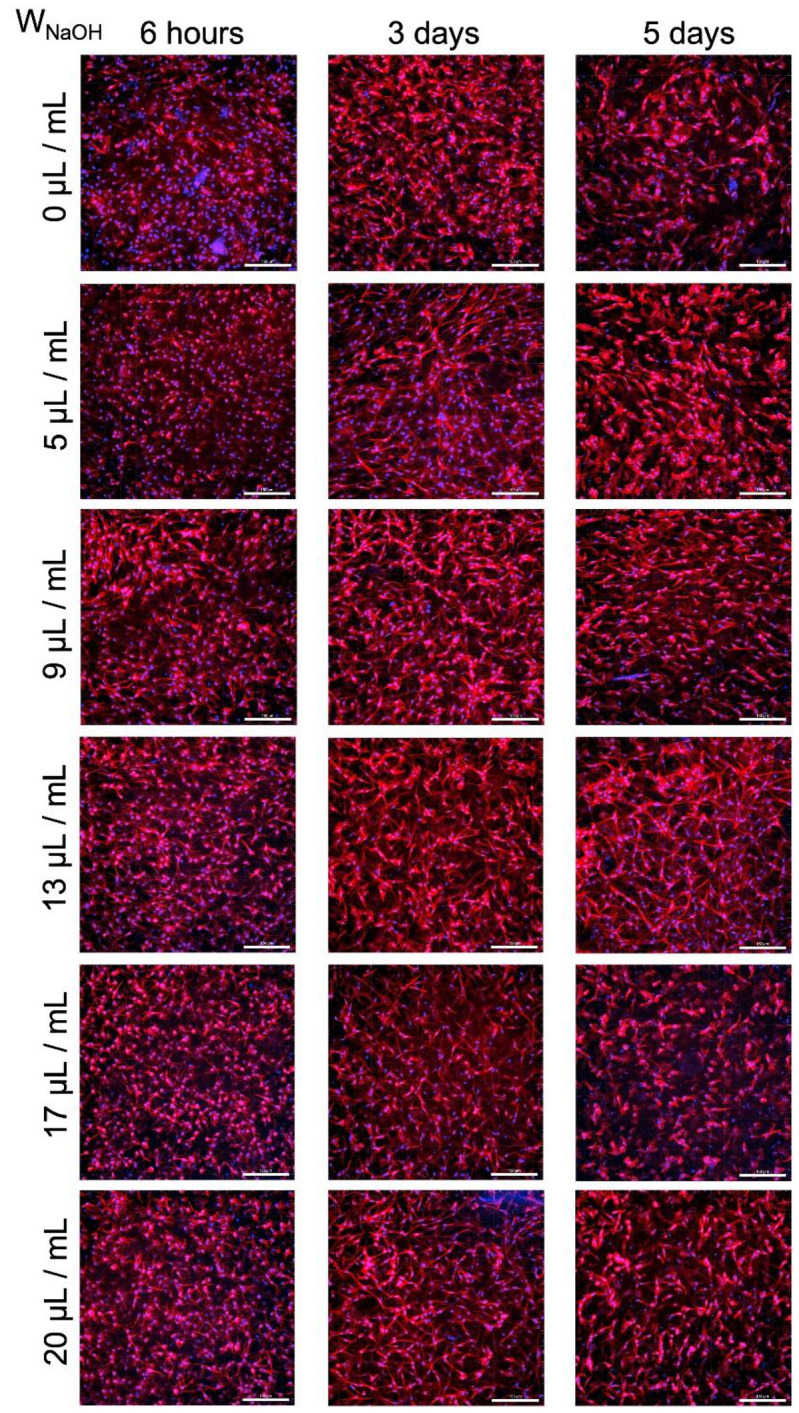
Comparison of the morphology of cells in samples with various NaOH concentrations after 6 h, and 3 and 5 days of cultivation. Cells in all samples were initially well grown, although lower cell densities were evident in sample 0. On the fifth day of cultivation, there were significant differences between the samples. F-actin in the cell cytoskeleton was stained with TRITC-conjugated phalloidin. Cell nuclei were counterstained with DAPI. Confocal microscope with spinning disc Nikon CSU-W1, objective CFI Plan Apo Lambda 20×, scale bar 150 μm.

DAPI/phalloidin staining was used to assess the morphology and spatial distribution of cells in the gels after 6 h, and 3 and 5 days of cultivation (Figure 8). At first glance, the reduced number of cells in the extremely acidic sample (0 μL/mL) was noticeable. The cells in the other samples formed a dense structure. With the increasing of the culture time, the cells, especially in samples 0, 17 and 20, were poorly elongated, round, and not interlaced. The cells of sample 5 formed a homogeneous structure, but on the fifth day of culture, they became round, with a granular structure. This phenomenon also occurred to a lesser extent in sample 9. In addition, isolated clusters of cells were found in samples 5 and 9 on the fifth day of cultivation. Sample 13 showed the best cell morphology throughout the culture period, with the best proliferation of all samples. The cell density was slightly lower on day 5 of culture for all samples.

**Figure 9:**
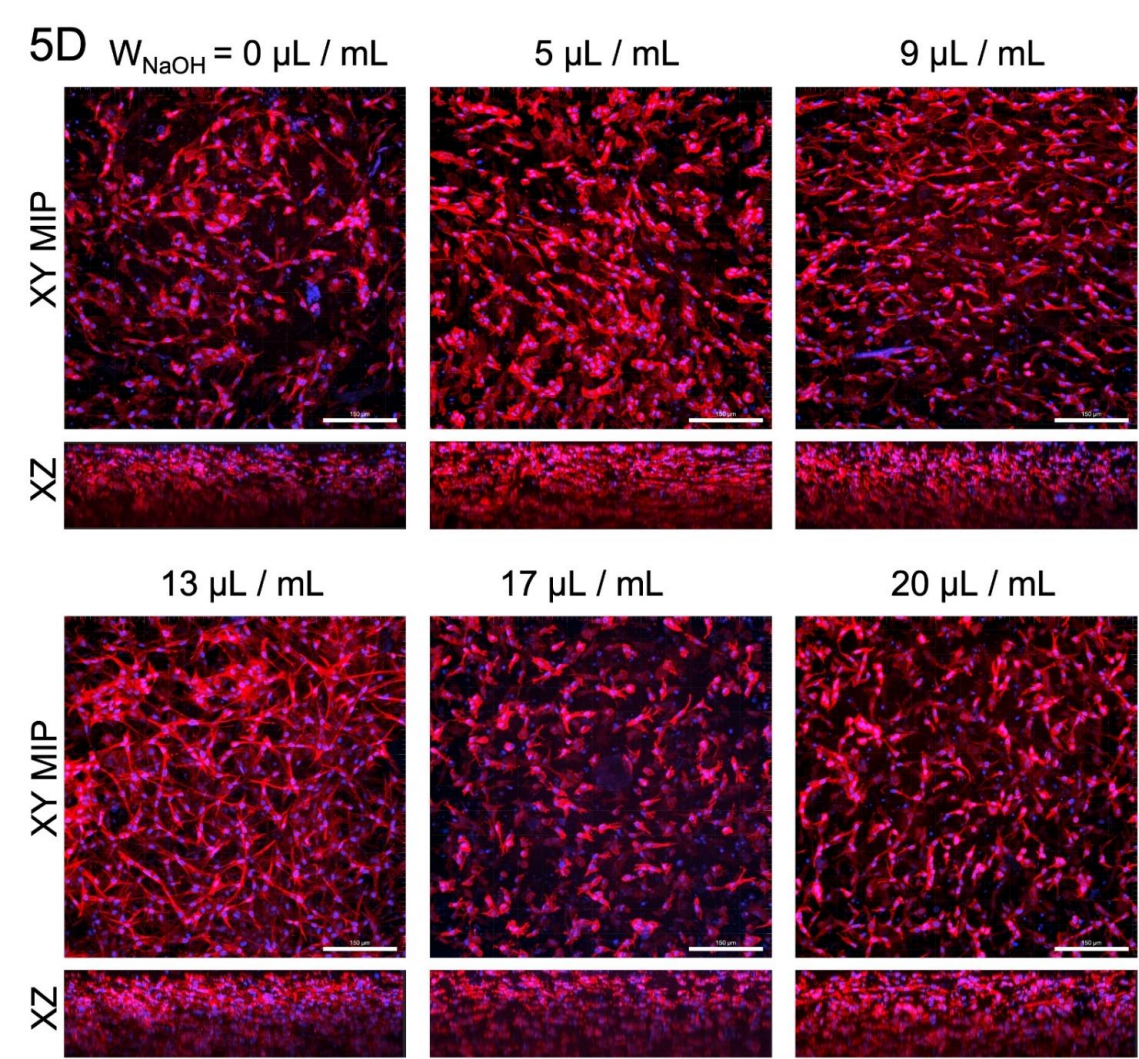
Detailed view of cell cultures of samples with various concentrations of NaOH, including view of the xz plane after 5 days of cultivation. Except for sample 0, cells were relatively homogeneously spread on the cross-sections. The cells were regularly distributed in all samples. F-actin in the cell cytoskeleton was stained with TRITC-conjugated phalloidin. The cell nuclei were counterstained with DAPI. Confocal microscope with spinning disc Nikon CSU-W1, objective CFI Plan Apo VC 20×, scale bar 150 μm.

Detailed sample images on fifth day of cultivation, including the view of the xz plane, are illustrated in Figure 9. Cells were spread throughout the gel volume. Gravity caused a dense layer of cells at the bottom of the gel (top of the xz-plane in the figures).

**Figure 10:**
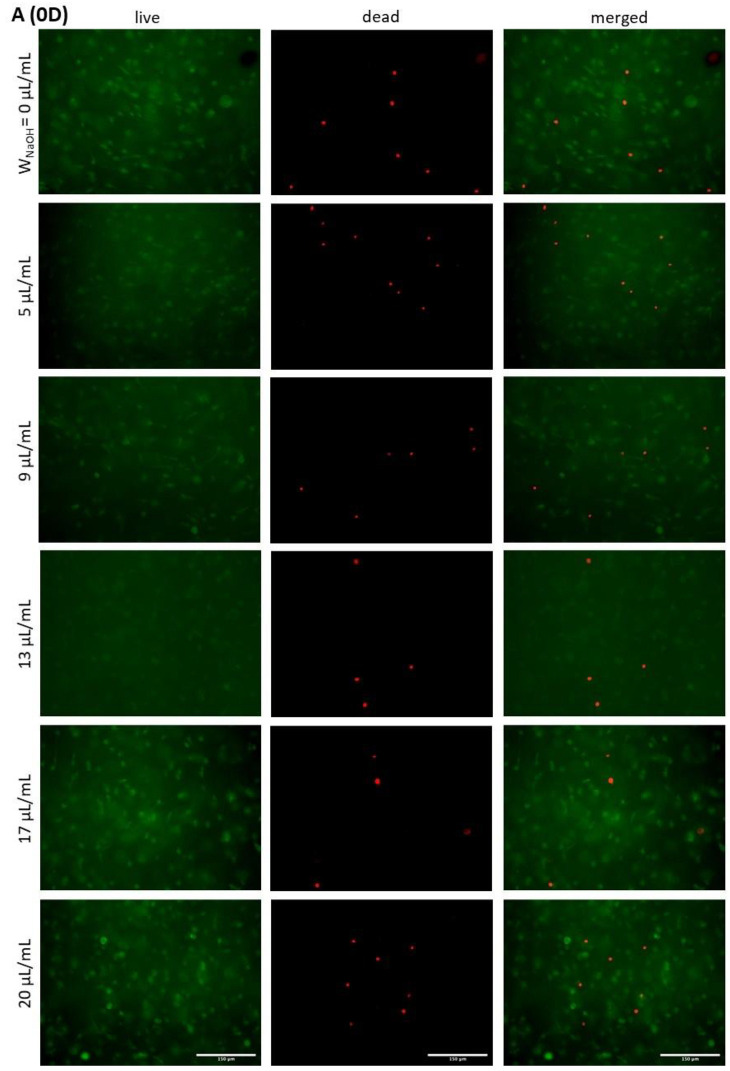

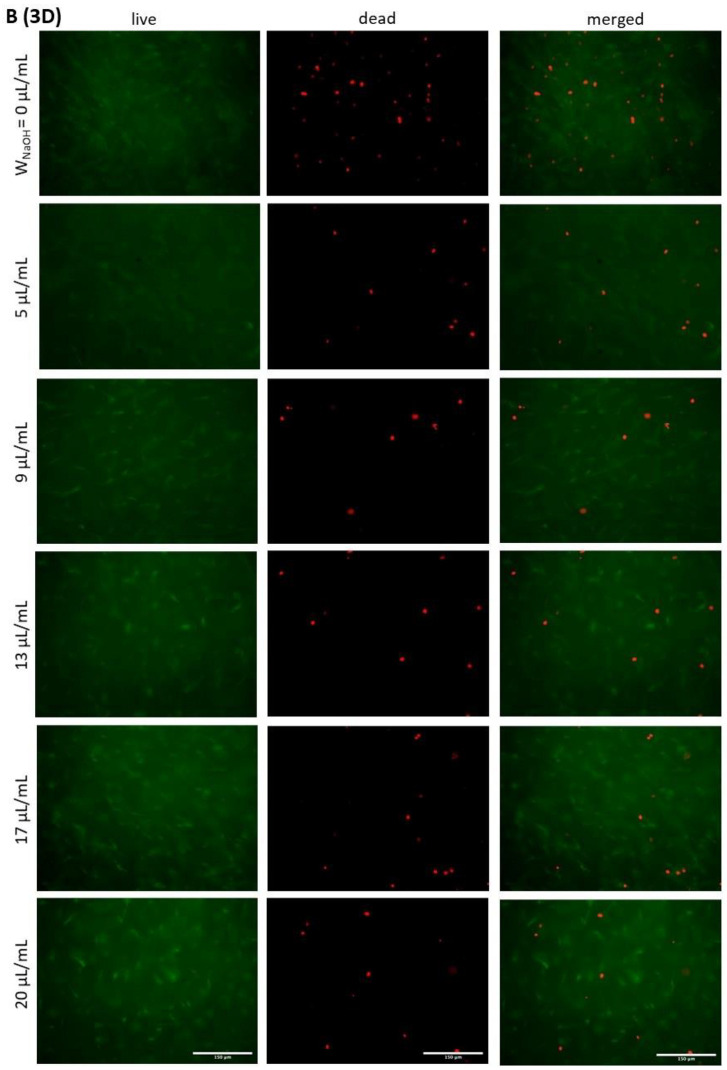

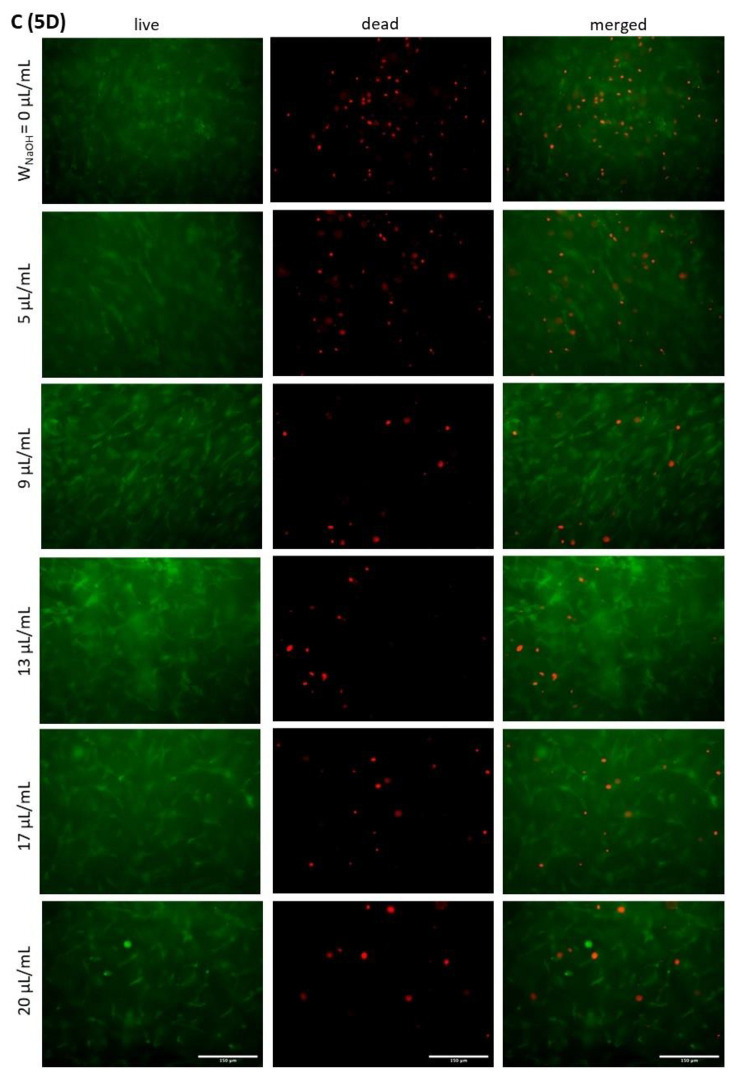
(**A**–**C**). The viability of cells in samples after 0 (6 h; (**A**)), 3 (**B**) and 5 (**C**) days of cultivation. Cell viability was examined using FDA/PI staining. Green cells were alive, and red cells were dead. Leica DMi8 microscope with N-Plan objective 20×, scale bar 150 μm.

**Figure 11:**
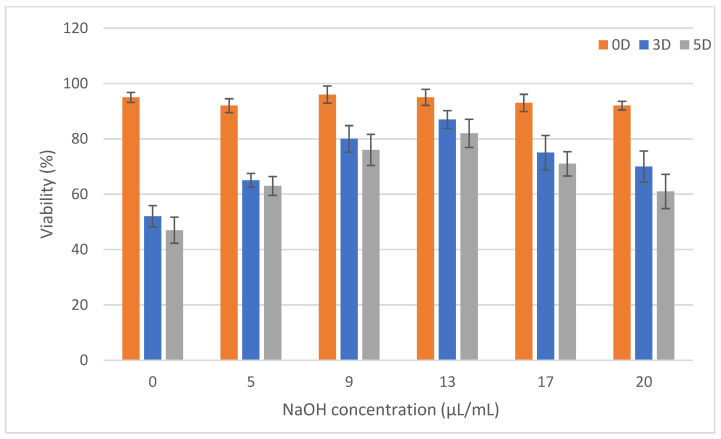
The viability of cells in samples after 0 (6 h), 3 and 5 days of cultivation. Data are represented as mean ± standard deviation. The data express the number of living cells as a proportion of the total number of cells. The numbers of cells were counted from pictures of live and dead cells (N = 10) at a 20-fold magnification.

Images of live/dead cells after 0 (6 h), 3 and 5 days of cultivation are shown in Figure 10, and the counted cell viability is shown in Figure 11. Measurements show that the cell viability, immediately after printing, was relatively high (above 90% for all samples), and that cooling of the bioink before printing did not have a large effect on cell survival. There was a large difference between the samples on the third and fifth day of cultivation. High numbers of live cells were measured in samples 9, 13, 17 and 20, resulting in overall high cell viability of the samples regardless of culture time. This was the case despite the fact that samples 17 and 20 had a relatively high number of dead cells. In contrast, samples 0 and 5 had relatively low numbers of live cells and high numbers of dead cells, resulting in viabilities of around 60 and 50%, respectively. Thus, the highest viability was observed in sample 13, which, however, was not significantly higher than samples 17 and 9. With a bias towards acidic or alkaline values, cell mortality increased. According to the results, it appears that an acidic environment is less favourable for cells than an alkaline environment, with cell viability being slightly higher at alkaline values.

There were occasional areas of mostly dead cells in the samples. This was due to irregularities in the gel structure, which are discussed in more detail below. These areas were not included in the overall viability of the sample.

It is known that the concentration of collagen and, therefore, the stiffness of the gel affects cell viability. In our work, we compared hydrogels where the concentration of collagen was identical, but the acidity of the gels was modified by the addition of NaOH. According to the literature, the optimal pH of the environment for cell growth is 7.38–7.87 [42]. Our prepared gels, immediately after mixing all components, reached values of 6.1 to 9.1. After crosslinking the gels in an incubator at 37 °C and in a 5% CO_2_ atmosphere, the values were adjusted to slightly basic values, from 7.6 to 8.2, due to the presence of buffers in the bioink components. The gels then became more acidic due to cell metabolism after 3 and 5 days of cultivation.

The acidity of the samples immediately after mixing proved to be a critical factor for cell survival and growth in the hydrogel. The overly high acidity of sample 0, which had a pH of 6.1, caused high cell apoptosis, yet the remaining cells were viable and survived until the fifth day of culture. As the pH increased, cell viability and overall morphology increased, with sample 13 showing a relatively dense cell culture with a cell viability greater than 80% on the fifth day of culture. This was also consistent with the assumptions made in the literature, as the pH of the 13 μL/mL sample was 7.6 after mixing and increased to 8 after 30 min in the incubator. However, further increases in pH had already resulted in a decrease in viability, as a well as worse cell morphology, which was particularly evident on the fifth day of culture.

We showed that the cells remained viable in all samples, but their viability depended on the pH of the structure. Thus, according to the above statement, it can be determined that the acidity of the environment has a greater influence on cell viability than the stiffness of the collagen gel itself.

### 2.5. Estimation of Bioink Mechanical Properties

**Figure 12:**
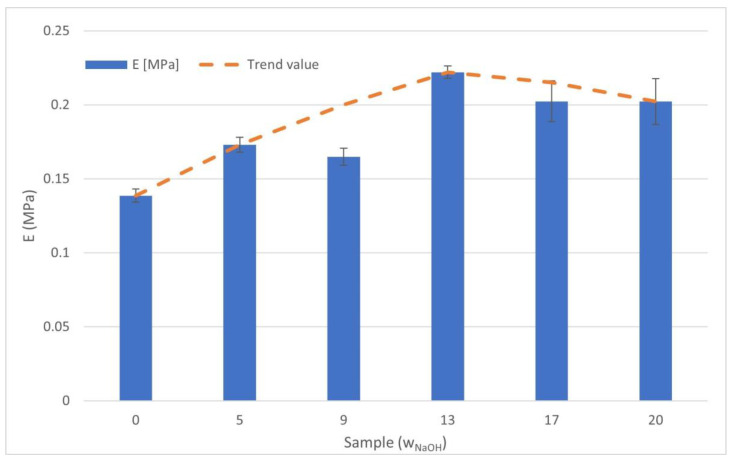
Compressive elastic modulus of prepared samples. Bars show the mean value with SD, N = 4, and the dashed line represents the trending value. A lower value of sample 9 μL/mL was possibly caused by the formation of microbubbles in the sample that resulted in a more compressible structure.

The compressive modulus was calculated for samples of all NaOH concentrations. The measured elastic modulus showed a characteristic pattern in which the value increased with increasing of the NaOH concentration up to a sample with 13 μL/mL, after which it started to decrease again, as shown in Figure 12. The highest value (13 μL/mL) was 0.2221 ± 0.0042 MPa, and the lowest value (0 μL/mL) was 0.1386 ± 0.0044 MPa. The orange dashed line on the graph shows the predicted trend of the values. The sample value of 9 μL/mL differed from this trend, resulting in a falsely lower value due to the inaccuracies described in the above discussion.

The compressive elastic modulus values we found were in the order of the compressive elastic moduli of other similar gel materials [43,44]. The results show that the material with 13% NaOH concentration had the highest elastic modulus value (E = 0.2221 ± 0.0042 MPa), while the material with zero NaOH concentration had the lowest value (E = 0.1386 ± 0.0044 MPa). From the measured results, it can be said that the compressive modulus value increased with the increasing of the NaOH concentration up to sample 13; then, up to sample 20, the modulus value decreased slightly. The exception to the trend described above was the material with a concentration of 9 μL/mL, where there was a slight local decrease in the value of the compressive modulus of elasticity compared to the trend. This was probably due to the inhomogeneity of the material caused by the presence of bubbles in the material. Despite this, the dependence of the trend of the elastic modulus dependence on NaOH concentration was clear.

The mechanical properties of hydrogels vary considerably in the literature, with the compressive modulus of collagen hydrogels ranging from tenths to tens of kPa. For example, in a study by Antoine et al. [9], compressive modulus values ranging from 0.5 to 11 kPa were found, depending on the pH, temperature and collagen concentration (4–10 mg/mL). In a study by Rhee et al. [27], it was reported that the compressive modulus of hydrogels with high collagen concentrations (20 mg/mL) takes values of about 30 kPa.

In view of this, it can be concluded that the compressive modulus values vary considerably across the literature. The mechanical properties of hydrogels depend on the composition of the hydrogel itself, the conditions under which the sample was prepared, and finally the conditions under which the sample was measured. The lack of a universal protocol that unifies both the production and the conditions for testing mechanical properties by compression testing affects the comparability of results across the literature.

In our work, we were not interested in the exact value of the compression modulus, but rather, were interested in the value of the relative change in the compression modulus, and the trend of this change, as a function of the sample preparation procedure.

**Figure 13:**
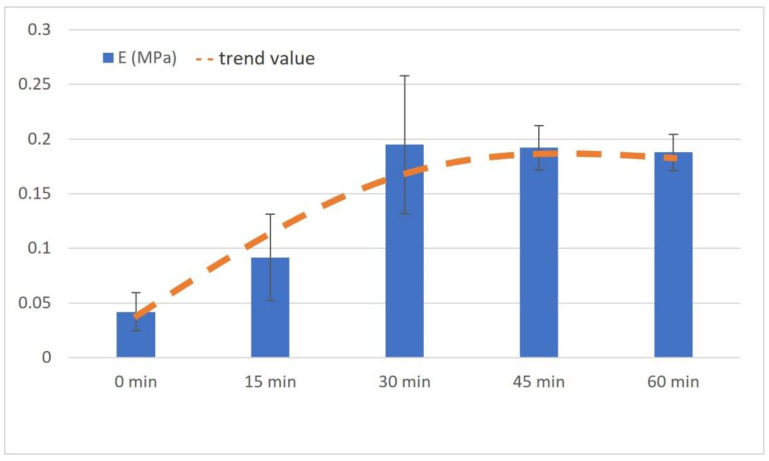
Compressive elastic modulus of samples with different gelling times. Bars show the mean value with SD, and the dashed line represents the trending value.

In chapter “2.3 The bioprinting procedure and sample form”, we mentioned that the gels were crosslinked in incubator for 30 min. This time was empirically set by measuring the compressive elastic modulus of the same samples with varying gelling/crosslinking times. As is illustrated in Figure 13, there was a major increase in compression modulus up to 30 min, then there was an equal or slightly decreasing trend for the compressive modulus.

### 2.6. Other Limitations

Type I collagen is widely used in the preparation of gels and scaffolds for chondrogenic tissues [45] as well as osteogenic [46], neural [32] or epithelial [47] tissues. In experimental studies, type I collagen-based tissues are implanted into patients to treat cartilage trauma conditions [48].

However, several studies have shown that the use of type I collagen causes cell dedifferentiation, as shown, for example, for chondrocytes in the study by Farjanel et al. [49]. The rapid expansion of chondrocytes in the monolayer leads to their dedifferentiation [50]. In addition, type III collagen was synthesized into type I collagen [50]. Therefore, the preparation of collagen gels for bioprinting must take this into account, and further research should be directed in this area.

Analysis of dead and living cells showed that there are places in the gels where most of the cells are dead. The cultivation of 3D scaffolds is practically limited by the thickness of the scaffold, with simple diffusion only transporting nutrients and oxygen, and removing of waste products, for structures up to a maximum thickness of 2 mm [51]. This is the case if the environment is homogeneous. In hydrogels with a collagen-type polymer, a higher density of fibres may be concentrated at one location, preventing diffusion, and the location is not optimally nourished. In bioprinted structures, there are also irregularities in gel shape, as described above, with some sites exceeding the 2 mm limit. In general, a vascular supply is required for tissues with a thickness greater than 2 mm [51], which is obviously not possible in the case of hydrogel scaffolds. However, the application of pressure loading in a dynamic culture system that promotes perfusion, cell proliferation and differentiation could improve this problem [52]. The mechanical stimulation of seeded hydrogels also helps in the remodelling of surrounding tissue by cells [53]. Further research on proliferation and differentiation in highly concentrated bioinks should be focused in this direction.

## 3. Materials and Methods

### 3.1. Isolation of Collagen

Type I collagen (COL), used to prepare the hydrogel matrix, was isolated from porcine skin using a 70% ethanol solution (*v*/*v*) (1 g skin/10 mL, 30 min), washed 3 times with water, and subsequently in an acetic acid solution at a ratio of 1:1000 (*v*/*v*) (1 g/20 mL, 48 h). COL in the collected supernatant was precipitated using a 0.1M NaOH solution at a 6:1 ratio (*v*/*v*) up to neutral pH. The obtained pellets were then dissolved in a 1:1000 (*v*/*v*) acetic acid solution, frozen to −30 °C and lyophilized. All isolates were stored in a freezer at −20 °C.

### 3.2. Analysis of Porcine Collagen Properties

The composition of isolated COL lyophilizate was analysed by various analytical methods. The determination of interstitial water (directly hydrogen bonded to triple-helix) [54] was performed according to the standard ISO 6496:1983 (Animal feeding stuffs–Determination of moisture content), i.e., drying to 160 ± 2 °C for 3 h.

Before amino acid (AA) analysis, the samples were hydrolysed in 6M hydrochloric acid at 110 °C/23 h, then vaporized and diluted in buffer. The amino acids containing sulphur were oxidised immediately before hydrolysis using peroxoformic acid at 5 °C/16 h. The analysis was performed using an Ingos AAA 400 analyser. The hydroxyproline (Hyp) content was determined according to the ISO 3496:1994(E) standard (Meat and Meat products–the determination of hydroxyproline content). The sample hydrolysate was oxidised using chloramine B. Following the quantitative reaction with p-dimethylaminobenzaldehyde, the hydroxyproline content in the hydrolysate was detected spectrophotometrically at 558 nm using a Unicam UV 530.

The lipid content was assessed using the Schmidt-Bondzyński-Ratzlaff method, according to the Czech technical standard EN ISO 1735:2004. The total content of glycosaminoglycans (GAGs, long unbranched polysaccharides) was quantified using the high-performance liquid chromatography (HPLC) method based on hexosamines (glucosamine and galactosamine) due to the formation of fluorescent N-acetylated hexosamine derivatives by reaction with a specific derivatization agent.

Polyacrylamide gel electrophoresis (SDS-PAGE) was performed using the Mini-Protean Tetra Cell electrophoretic system from BIO-RAD on TGX Miniprotean Precast Gel, 4–15% (BIO-RAD), with a separation voltage of 200 V applied for 30 min. A volume of 25 μL of COL in acetic acid solution was loaded into 1 well (10 μL in standard markers of molecular weight). Coomassie Brilliant Blue-R was used as the dye for the detection of the bands of the separate components.

The secondary structure of the isolated COL and the COL submitted to ethanol sterilization was evaluated by attenuated total reflection infrared spectrometry (ATR-FTIR) using an iS50 infrared spectrometer (Nicolet Instrument, Madison, WI, USA), the ATR device was equipped with a diamond crystal. All the spectra were recorded in absorption mode in the range 4000–400 cm^−1^ at a resolution of 4 cm^−1^ and 64 scans. Infrared spectra were worked on and evaluated using the OMNIC version 9 software.

### 3.3. Bioink Preparation and Cultivation Conditions

Lyophilized COL I was sterilized by immersion in 100% ethanol for 1 h. After removal from ethanol, the COL was freely dried in a laminar flow box. The lyophilized COL I was dissolved in 0.02 M acetic acid at a concentration of 20 mg/mL and stored at 4 °C for 10 days. The COL suspension was homogenized using a disintegrator (10,000 rpm, 1 min).

The production of the own collagen bioink was based on the procedures recommended by the Ibidi GmbH company [55] (Ibidi GmbH, Gräfelfing, Germany). Briefly, 20 mg/mL stock collagen hydrogel was diluted and neutralized by 10× concentrated DMEM culture medium (D5030, Sigma-Aldrich, St. Louis, MO, USA; supplemented by 10 g/L glucose, 5.84 g/L L-glutamine and 37 g/L sodium bicarbonate); 1× concentrated growth medium consisting of a 1:1 mixture of Low-Glucose (1 g/L) Dulbecco’s Modified Eagle Medium and Ham’s F-12 Medium (DMEM/F12, both Sigma-Aldrich; DMEM was supplemented by 1 g/L glucose, 0.584 g/L L-glutamine and 3.7 g/L sodium bicarbonate, F12 supplemented by 1.176 g/L), with 10% fetal bovine serum, 1% ABAM antibiotics (100 IU/mL of penicillin, 100 µg/mL of streptomycin, and 0.25 µg/mL of Gibco Amphotericin B; Sigma-Aldrich, St. Louis, MO, USA) and 10 ng/mL FGF2, deionized water, NaOH and cell suspension in 1× culture medium.

To optimize the pH of the bioink, a series of concentrations at 0, 5, 9, 13, 17 and 20 μL/mL of 1molar NaOH solution (the samples were then indicated in the text only by numbers without units) was created. This step aimed to find the optimal concentration and, therefore, pH for cell growth in concentrated collagen hydrogels. The volume of the hydroxide solution was subtracted from the volume of water added, so that the resulting volume always remained identical. The final pH was measured with a flat surface glass touch electrode (Theta 90, Prague, Czech Republic) immediately after mixing and after stiffening of the gel (37 °C, atmosphere with 5% CO_2_, 30 min). These resulted in six types of samples with different pHs, which were further analysed.

A cell suspension was prepared from porcine stromal cells derived from porcine adipose tissue isolated from the fat of the neck area. The whole procedure, including cell characterization, is described in a previous article by Matejka et al. [56]. Cells were pre-cultured in the growth medium described above in 75 cm^2^ culture flasks (TPP, Trasadingen, Switzerland). The third cell passage was used to make a suspension. Cell passaging was performed using a 1 mM EDTA solution and a 0.05% trypsin/0.5 mM EDTA solution in PBS when cells reached 90% confluence. To prepare bioink, cell suspension with a cell density of 8 mil/mL was prepared in a growth medium at a temperature of 10 °C.

All components were sterilised and cooled to a temperature of approximately 4 °C. First, collagen hydrogel was mixed with all components except cell suspension using two sterile syringes with Luer-lock coupling. Collagen was mixed with 10x culture medium, H_2_O, 1× concentrated culture medium and NaOH at the following ratios: 1:0.14:0.16–0.2:0.22:0–0.04. After neutralization, 0.44 of the previous part of the cell suspension was added and mixed using the next syringe. The ratio of components in the bioink is also shown in Figure 11. Due to the 50% collagen content of the bioink, our final collagen concentration was 10 mg/mL. Immediately after mixing, the syringe with the bioink was placed in the cooled bioprinter holder and printing was started. The printing process was carried out under sterile conditions in a laminar flow box.

The 8 × 2 mm discs were printed on a 24well plate, quadruplets of each sample were prepared for statistical analysis. The printed discs were transferred to a laboratory incubator (37 °C, 5% CO_2_) to stiffen for 30 min, then 1 mL of growth culture medium was added. The samples were cultivated under static conditions. On each third day, the culture medium was changed. Samples were fixed after 6 h, and 3 and 5 days of culture for further analysis.

### 3.4. Bioprinting of Samples

The printing of samples was performed using a modified Inkredible + bioprinter (CELLINK, Boston, MA, USA). The modification included a custom extruder and printing platform. The custom extruder used a linear actuator with a common Luer-Lock syringe as a cartouche with a water-cooled fixture. The benefit of this solution over pressure-controlled extrusion was precise control over the extruded volume. The printing platform contained a vacuum fixture to fix microscopic glass (26 × 76 × 1 mm^3^) in place and a water heating circuit. Temperatures were set at 10 °C for the extruder and 37 °C for the printing platform.

**Figure 14:**
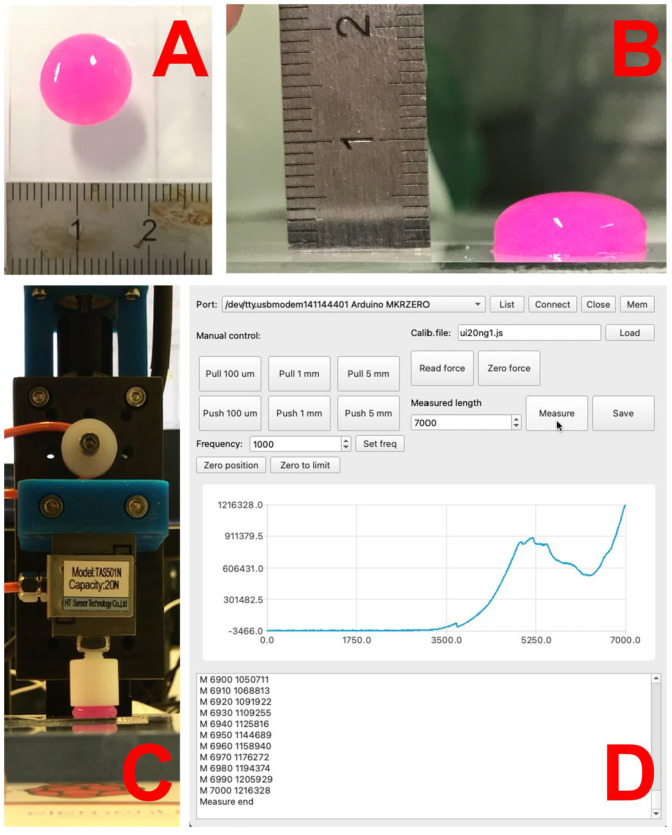
Printed bioinks on microscope glass: (**A**) top view; (**B**) side view. Estimation of mechanical properties: (**C**) custom testing device with linear actuator and force sensor; (**D**) custom control and acquisition software.

Two cylindrical models were used: for cell experiments, a cylinder with diameter of 8 mm and heightof 2 mm, and for the estimation of mechanical parameters, a cylinder with diameter of 12 mm and height of 5 mm. These models were sliced by hand, creating a custom G code for the printer. Briefly, the height of the layers was set to 0.5 mm. The diameter of the cylinders was sectioned into 1.5-mm thick circles without overlapping. The dispensing volume for each circle printed was set as the diameter of the circle with a 1.5 mm thick line with 0.5 mm height. A flat end stainless steel needle, of size G17 (approx. 1 mm), was used as a printing nozzle. The printing speed due to different gelling times was variable: 5 mm/s for 0, 5 and 9 μL/mL W_NaOh_; 3.5 mm/s for 13 μL/mL; 2.5 mm/s for 17 μL/mL; and 1.5 mm/s for 20 μL/mL. The printed sample is illustrated in Figure 14.

### 3.5. Cell Morphology, Cell Count, and Viability Assay

Immunofluorescence pictures of cells with DAPI-counterstained cell nuclei and F-actin cytoskeleton counterstained with TRITC-conjugated phalloidin were further utilized to evaluate cell spread, shape and morphology after 3 days of cultivation of the bioinks tested. Cells were fixed with 99.8% ice cold methanol (Sigma-Aldrich, St. Louis, MO, USA) for 20 min at −4 °C. To block nonspecific binding sites for antibodies, the samples were treated for 20 min at room temperature with PBS containing 0.1% Tween 20 (PBST), 1% bovine serum albumin (BSA), and 22.52 mg/mL of glycine.

Cells were then exposed to Phalloidin-conjugated tetramethylrhodamine (TRITC), which stained filamentous actin (F-actin) in the cell cytoskeleton (Sigma-Aldrich, Cat. No. P1951, 2 µg/mL) and DAPI (Thermo-Fisher, Waltham, MA, USA; D1306, 300 nM concentration) at RT for 120 min in humidified chambers. The samples were then rinsed and stored in PBS at 4 °C. These microscopic images were taken using a Nikon CSU-W1 inverted spinning disc confocal microscope based on the Nikon Eclipse Ti2 inverted microscope (Nikon, Tokyo, Japan) with a Yokogawa CSU-W1 spinning disc module (Yokogawa, Tokyo, Japan) and equipped with dual sCMOS PRIME BSI cameras (Teledyne Photometrics, Tucson, AZ, USA). Due to the thickness of the samples, Nikon’s dry objectives CFI Plan Apo Lambda 10X and CFI Plan Apo VC 20x were used with a pinhole disc of 25 μm in Z stack mode (350–400 μm depth). The images were then processed in Imaris software (Oxford Instruments, Abingdon-on-Thames, Great Britain). Processing involved the maximum intensity projection in the XY plane (MIP XZ) and the maximum intensity projection in the XZ plane (MIP XZ) to visualize pseudo sections of sample volume. To enhance contrast at lower levels of the Z stack planes, the gamma correction was set to 2.2 for the red channel (phalloidin) and 1.5 for the blue channel (DAPI).

Cell viability was identified by live cell staining with fluorescein diacetate (FDA, 5 mg/mL; F1303, ThermoFisher, Waltham, MA, USA) and dead cell staining with propidium iodide (PI, 2 mg/mL; P1304MP, ThermoFisher, Waltham, MA, USA) after 6 h, and 3 and 5 days of cultivation. The samples were rinsed three times with PBS and stained with FDA and PI diluted in culture medium without fetal bovine serum for 20 min at 37 °C in a humidified incubator. According to the manufacturer’s instructions, the live and dead cells were labelled green and red. The images were then taken using the Leica DMi8 wide-field fluorescence microscope with N-Plan 5 × 1 10× and 20× objectives (Leica, Wetzlar, Germany) and the DCU223M CCD camera (Thorlabs, Bergkirchen, Germany). Images of 10 randomly selected microscopic fields were taken. The resulting viability of the sample was then assessed as the number of live cells compared to the total number of cells in the field. The resulting rank was the average of the counted fields.

### 3.6. Estimation of Bioink Mechanical Properties

The samples were tested by compression test, which appears to be a suitable method for determining the mechanical properties of gels [43]. The test was carried out using the custom laboratory-made testing device. This device consists of three main parts. Mechanical movement to generate compression of printed bioink is realized by a linear actuator platform with 0.5 mm pitch micro screw actuated by the stepper motor. This platform has a 20 N force sensor TAS501N (HT Sensor Technology, Xi’an, China) with a 15 mm PTFE pushing disc. The movement of the stepper motor and the acquisition of the analog signal are performed using an ARM-based Arduino Zero MKR (Arduino LLC, Somerville, MA, USA) with custom software made on the Qt platform (QtGroup, Espoo, Finland).

The sample was compressed between two parallel surfaces while the deformation was controlled and the force was recorded. The sample was loaded until its destruction. The device’s zero height was set to be 7.3 mm above the microscopy glass where the sample was printed (2 mm above the top of the printed sample for safe manipulation). The compression movement was set at a constant speed of 0.5 mm/s with a travel distance of 7 mm (0.3 mm above the microscopic glass surface).

Using this method, the dependence of force on the deformation was obtained. Then, the slope of the linear part of the force–deformation curve gave us the value of measured material stiffness. The stiffness of a material was proportional to its compressive modulus of elasticity, so it was relatively easy to convert the stiffness of a material to its compressive modulus of elasticity.

(1) $$E=\frac{k\cdot l_{0}}{A},$$

where *l*_0_ is the initial height of the sample, *k* is the stiffness of the sample, and *A* is the cross section of the sample.

Initially, it was assumed that the samples would be cylindrical. Unfortunately, this could not be fully achieved because printing technology was used to produce the samples.

Compared to the originally intended cylinder (or disk), the top of the disk, due to surface tension, was rounded into a drop shape. However, this change in shape was negligible, and therefore, the shape of the sample was considered a cylinder. The radius of the sample was measured using a microscope and the height was calculated from the volume of material used for sample production.

## 4. Conclusions

In this study, we showed that it is possible to print highly concentrated collagen hydrogels with incorporated cells, where the viability of the cells in the gel remains very good. This was found to be achievable simply by optimizing the properties of the bioink, particularly the gel composition and pH modification, as well as by optimizing the printing parameters. The maintenance of the bioink temperature at 10 °C before printing followed by heating to 37 °C after extrusion, as well as adjusting the printing speed, was found to be crucial. Bioink made of collagen hydrogel was tested with an initial collagen concentration of 20 mg/mL. With a final collagen concentration of 10 mg/mL, these collagens were considered unsuitable for cell growth, as the cells had low proliferation. However, our results showed that a balanced bioink composition could be achieved with pH adjustment by adding the optimal amount of NaOH (13 μL per mL of bioink allowed cells to grow and divide). This modification also contributed to a higher elastic modulus of the hydrogel, which reached 0.2221 ± 0.0042 MPa, allowing printing of structures up to several millimeters in size with sufficient mechanical resistance. With this experiment, we showed that a high concentration of collagen gels may not be a limiting factor for cell proliferation. However, by optimizing the composition and acidity of the bioink and proper printing settings, satisfactory results could be achieved.

## Figures and Tables

**Table 1:** Composition of COL lyophilizate isolated from porcine skin. “*” represents the lower and upper quartiles.

Amino Acid	Numbers of AA Residues/1000 Units	Amino Acid	Numbers of AA Residues/1000 Units
Asp + Asn	49	Cys	2
Glu + Gln	77	Met	6
Thr	17	Tyr	3
Ser	32	Phe	14
Gly	320	Lys	31
Ala	110	His	15
Val	31	Arg	51
Ile	11	Pro	122
Leu	27	Hyp	82
Protein (wt%)		59.20	
Hyp (wt%)		5.63 (4.95–6.03) *	
Degree of hydroxylation (%)		40	
GAGs (wt%)		4.24 (3.12–5.96) *	
Lipids (wt%)		23.98 (21.49–31.27) *	
Water (%)		7.17 (6.99–7.29) *	
